# Adenovirus 36 DNA in Adipose Tissue of Patient with Unusual Visceral Obesity

**DOI:** 10.3201/eid1605.091271

**Published:** 2010-05

**Authors:** Behrouz Salehian, Stephen J. Forman, Fouad R. Kandeel, Denise E. Bruner, Jia He, Richard L. Atkinson

**Affiliations:** City of Hope and Beckman Research Institute, Duarte, California, USA (B. Salehian, S.J. Forman, F.R. Kandeel); Denise Bruner and Associates, Arlington, Virginia, USA (D.E. Bruner); Obetech Obesity Research Center, Richmond, Virginia, USA (J. He, R.L. Atkinson)

**Keywords:** Visceral obesity, adenovirus 36, adipose tissue, lipoma, DNA, PCR, viruses, dispatch

## Abstract

Massive adipose tissue depositions in the abdomen and thorax sufficient to interfere with respiration developed in a patient with multiple medical problems. Biopsy of adipose tissue identified human adenovirus 36 (Adv 36) DNA. Adv 36 causes adipogenesis in animals and humans. Development of massive lipomatosis may be caused by Adv 36.

Infection with human adenovirus 36 (Adv 36) has been reported to cause a large accumulation of fat in 4 animals (chickens, mice, rats, and monkeys) (*1**–**3*). Selective deposition of visceral fat disproportional to total fat deposition was observed in some studies. The increase in visceral fat or total body fat in infected animals compared with uninfected animals was >100% in some experiments (*1**–**3*). Of animals that were infected, 60%–100% became obese compared with uninfected animals (*1**–**4*). Obesity was defined as a weight or fat content greater than the 85th percentile of the uninfected animals.

Several human studies have shown a correlation of antibodies to Adv 36 and obesity (*4**–**8*). In 1 study of >500 persons, 30% of obese persons and 11% of lean persons had antibodies to Adv 36 (*4*). The body weight of infected persons was ≈25 kg heavier than that of uninfected persons (*4*). In 26 pairs of twins with discordant Adv 36 antibody status, infected twins were heavier and fatter (*4*). In a group of obese school children from South Korea, 30% had antibodies to Adv 36, and infected children had higher body mass index z-scores than uninfected children (*5*). However, in animals and adults in the United States, serum cholesterol and triglyceride levels were paradoxically reduced, despite the obesity (*1**–**4*). Recent reports of adults in Italy and children in South Korea support the association of Adv 36 and obesity, and show that Adv 36 is more common in obese persons; prevalence ranges from 29% to 65% (*6**,**7*).

The mechanisms responsible for the increased adiposity are changes in gene expression of multiple enzymes and transcription factors by the virus (*8**–**15*). In adipocytes, the sterol regulatory element binding protein pathway is increased, resulting in increases in levels of sterol regulatory element binding protein 1 and fatty acid synthase. Because levels of transcription factor CCAAT/enhancer binding protein-β, peroxisome proliferator-activated receptor-γ, and lipoprotein lipase are also increased, lipid transport into cells and fatty acid synthesis within cells is increased (*8**–**15*). In muscle cells, gene expression of glucose transporters Glut 1 and Glut 4 and phosphoinositide 3-kinase is increased, which results in noninsulin-mediated increases in glucose transport (*14*).

These changes are thought to be caused by the action of the Adv 36 open reading frame 1 early region 4 gene and may be blocked by small interfering RNA or the antiviral drug cidofovir (*11**,**13*). When the open reading frame 1 early region 4 gene was transferred to a retrovirus and inserted into preadipocytes in vitro, the gene was capable of inducing the enzymes and enhancing fat accumulation (*13*).

Adv 36 DNA persists in multiple tissues of infected animals for long periods after initial infection (*3*). Viral DNA was isolated from brain, lung, liver, muscle, and adipose tissue of monkeys 7 months after initial infection, long after the active virus has disappeared from blood and feces (*3*). The virus DNA apparently continues to alter gene expression chronically in tissues.

We report a patient with massive fat deposits in the thorax and abdomen. We postulate that these abnormal adipose tissue deposits were caused by Adv 36 infection.

## The Patient

The patient, a 62-year old man who was diagnosed with high-grade large cell lymphoma in 1999, received multidrug (cyclophosphamide, doxorubicin, vincristine, and prednisolone) chemotherapy, central nervous system prophylaxis with cytarabine, and high-dose methotrexate. In February 2000, he underwent autologous bone marrow transplantation and received a conditioning regimen of etoposide, cytoxan, and fractionated total-body irradiation. Hypothyroidism, chemoradiation-induced hypogonadism, and adrenal insufficiency developed in the patient, which required chronic glucocorticoid replacement.

**Figure 1:**
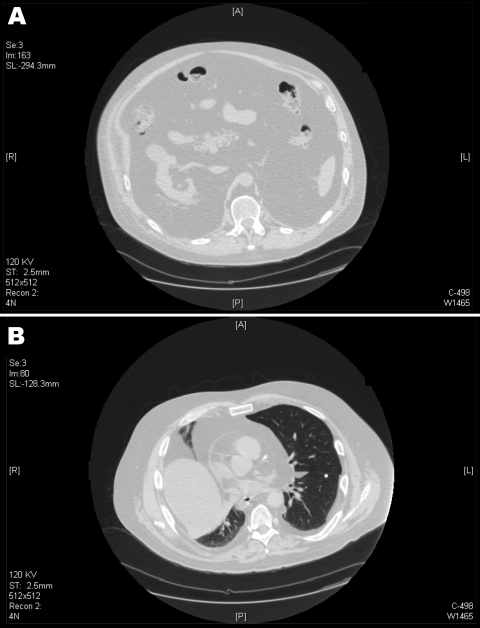
Computed tomography scans of the patient, showing marked visceral adipose tissue in the abdomen (A) and thorax (B). Diffuse intrabadominal, retroperitoneal lipomatosis, and herniation of the mediastinum can be seen through the esophageal hiatus. Intrapericardial adipose infiltration and adipose tissue bilaterally are seen within the pleura.

During the next 7 years, prostate cancer, rectal ulcer necessitating colon diversion, hemolytic anemia, thrombocytopenia, myelodysplastic syndrome, and diabetes mellitus developed in the patient; he was treated with insulin for the diabetes. He was hospitalized for respiratory insufficiency in August, 2007, which was thought to be caused or exacerbated by massive intrathoracic and intraabdominal fat deposits. He had obesity of his neck, lateral chest, and abdomen, but limited subcutaneous fat in his abdomen and upper extremities. He had no buffalo hump, round facies, or other signs of Cushing syndrome. The patient had tachycardia with muffled heart sounds, dullness in the base of the right chest, and bibasilar diminished breath sounds. A computed tomography scan of the chest and abdomen showed fatty densities extending into the intrabdominal, intraperitoneal, and retroperitoneal areas and herniating through the esophageal hiatus into the mediastinum (Figure 1). These fatty densities extended within the pericardium without definite pericardial effusion.

The patient’s weight was 113 kg, height 183 cm, body mass index 34, waist circumference 145 cm, and hip circumference 111 cm. Laboratory tests showed triglycerides 1.356 mmol/L, total cholesterol 2.2015 mmol/L, high-density lipoprotein cholesterol 0.5957 mmol/L, and low-density lipoprotein cholesterol 0.9842 mmol/L. The serum lipids values represent marked decreases from previous measurements. In December 2002, his low-density lipoprotein cholesterol level was 2.7412 mmol/L. In April 2007, his serum triglyceride level was 4.92244 mmol/L. The result of a test for serum immunoglobulin (Ig) M against adenoviruses was negative (0.07 IU), and the result of a test for serum IgG was positive (2.18 IU).

**Figure 2:**
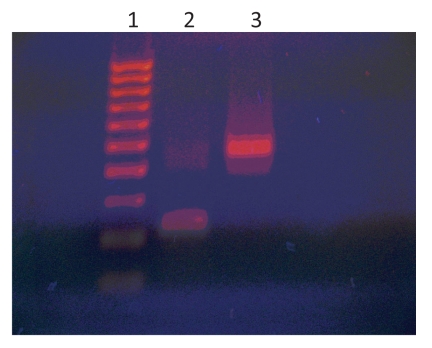
*Hae*III digestion of adenovirus 36 (Adv 36) DNA PCR products of the patient. Lane 1, molecular size marker; lane 2, *Hae*III digest of Adv 36 DNA; lane 3, undigested Adv 36 DNA.

Infection with Adv 36 causing disseminated lipomatosis was suspected. A subcutaneous fat biopsy specimen was assayed for Adv 36 DNA by nested PCR (*4*). Three of 4 adipose tissue samples showed a band compatible with Adv 36 DNA. Water controls in the assay had negative results. A *Hae*III digest of the presumed Adv 36 DNA band showed digestion at the expected site and yielded 2 bands of equal size (Figure 2). Sequencing of the DNA band by the Virginia Commonwealth University Massey Cancer Center Molecular Biology Core (Richmond, VA, USA) identified the sequence as Adv 36 DNA.

As a control, samples of adipose tissue obtained by needle fat biopsy from 12 obese persons without abnormal adipose tissue deposits were evaluated by nested PCR and quantitative PCR by using proprietary Taqman primers and probe (Obetech, Richmond, VA, USA). These persons provided written informed consent. Samples for quantitative PCR were analyzed with an ABI Step One PCR apparatus (Applied Biosystems, Foster City, CA, USA). Two of the 8 samples assayed by nested PCR were positive and 5 of 12 samples assayed by quantitative PCR were positive. The prevalence of Adv 36 infection identified by PCR was similar to that identified by serum neutralization in obese adults in the United States (*5*).

## Conclusions

Adv 36 DNA in the adipose tissue of this patient documents that he was infected with this virus. The propensity of Adv 36 to increase visceral adipose tissue in experimentally infected animals suggests that the abnormal adipose tissue deposits within the abdomen and chest cavities and in the subcutaneous spaces of the chest and neck could be caused by Adv 36 infection. He was being treated with replacement corticosteroids but did not have signs of Cushing syndrome.

More research is needed to determine if Adv 36 plays a role in abnormal adipose tissue deposits/lipomatosis. If Adv 36 is found to be a cause, research is needed to identify effective antiviral agents with a more tolerable side effect profile. Cidofovir is effective against Adv 36 in vitro, but has major side effects in humans.
